# Connecting electrodes with light: one wire, many electrodes†
†J. J. G conceived the project, analyzed data and prepared the manuscript. M. C. H and S. C. performed the majority of the experiments, analyzed the data and contributed to discussing and editing the manuscript. Y. Y. performed anthraquinone experiments, Y. Z. fabricated the patterned surfaces, R. T., L. Z. and V. R. G. contributed to performing the DNA experiments.
‡
‡Electronic supplementary information (ESI) available: Synthetic methods, additional details on the optical set up, characterization of the interfaces by XPS and electrochemically plus preliminary results on the polypyrrole and DNA experiments. See DOI: 10.1039/c5sc03011k



**DOI:** 10.1039/c5sc03011k

**Published:** 2015-08-28

**Authors:** Moinul H. Choudhury, Simone Ciampi, Ying Yang, Roya Tavallaie, Ying Zhu, Leila Zarei, Vinicius R. Gonçales, J. Justin Gooding

**Affiliations:** a School of Chemistry , The University of New South Wales , Sydney , NSW 2052 , Australia . Email: justin.gooding@unsw.edu.au; b Australian Centre for NanoMedicine , The University of New South Wales , Sydney , NSW 2052 , Australia; c ARC Centre of Excellence in Convergent Bio-Nano Science and Technology , The University of New South Wales , Sydney , NSW 2052 , Australia

## Abstract

The requirement of a wire to each electrode is central to the design of any electronic device but can also be a major restriction. For example it entails space restrictions and rigid device architecture in multi-electrode devices. The finite space that is taken up by the array of electrical terminals and conductive pads also severely limits the achievable density of electrodes in the device. Here it is shown that a travelling light pointer can be used to form transient electrical connections anywhere on a monolithic semiconductor electrode that is fitted with a single peripheral electrical terminal. This is achieved using hydrogen terminated silicon electrodes that are modified with well-defined organic monolayers. It is shown that electrochemical information can be either read from or written onto these surfaces. Using this concept it is possible to form devices that are equivalent to a conventional electrode array but that do not require a predetermined architecture, and where each element of the array is temporally “connected” using light stimulus; a step change in capability for electrochemistry.

## Abstract 2

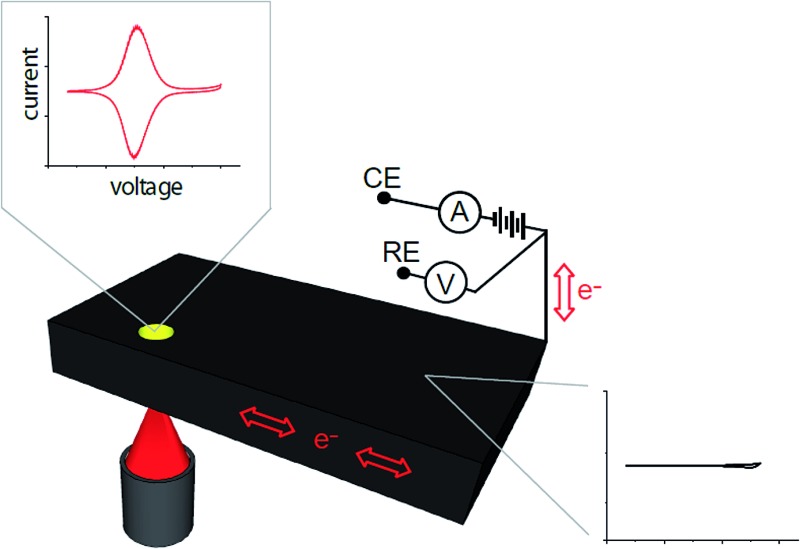
The requirement of a wire to each electrode is central to the design of any electronic device but can also be a major restriction. Herein it is shown how electrodes can be connected using light such that a multielectrode device requires only a single physical wire.

## Introduction

A central tenet of electrochemistry is that any electrode must be connected to an external circuit *via* a wire such that a potential can be applied to the electrode or the electrode potential can be monitored. Implicit in this central tenet is that for multiple electrodes that operate independently in an electrode array, there must be a wire connecting each element of the array.^1^ There are two limitations to this requirement. First, the connecting wires and associated bonding pads use considerable space on a chip surface which restricts the density of electrode that can be in an array such that the density of elements in an electrochemical array cannot match what is achievable with optical arrays. The second consequence is that the position of each micro-fabricated conductive feature in an array must be pre-defined. That is, the geometry of the array is intrinsically restricted to a choice made by the experimentalist prior to the experiment. Strategies to partially address the first of these limitations have been reported by forming columns and rows of electrodes in a grid pattern.^2,3^ As electrochemistry is confined to the crossover points of the columns and rows so that contacts are shared. Using such an approach, Kelley and co-workers^2^ showed only 30 contacts are required to connect 100 electrodes. However, a conceptual solution to address the requirement for preorganization, has yet to be proposed.

Other groups have demonstrated strategies that can free electrodes from the restriction of individual connecting wires. Arguably, the best known of these is scanning electrochemical microscopy (SECM).^4,5^ In a typical SECM set up an ultramicroelectrode interrogates the electrochemical behaviour of an electrolyte/electrode interface over a region of interest. Also very relevant to this work is the working principle of a light-addressable potentiometric sensors (LAPS). A LAPS device requires a single wire, an electrolyte–insulator–semiconductor interface and it is free of any 2D structuring. LAPS use AC photocurrents generated within the substrate to detect changes in the capacitance of the semiconductor depletion layer at the site of illumination. LAPS remain, however, chemical sensors with no DC currents crossing the interface.^6–9^ This is because an insulating layer, for example a silica layer on a silicon electrode, is necessary to stabilize the measuring interface as unmodified silicon is not stable in aqueous media. Stabilising silicon in aqueous media without an intervening oxide layer has been an enduring challenge.^10^

We have recently reported a solution to the elusive combination of surface chemistry that prevents oxidation of oxide free silicon but which is sufficiently thin that appreciable electron transfer occurs.^11^ Hydrogen-terminated silicon was first modified with a monolayer of a α,ω-dialkyne, 1,8-nonadiyne. One end of the molecule covalently attaches to the Si–H surface *via* hydrosilylation of the alkyne, as first described by Linford and Chidsey.^12^ The distal end of the monolayer then possesses an alkyne to which a redox active species, ferrocene was attached *via* the copper-catalyzed azide–alkyne cycloaddition (CuAAC) reaction. With a highly doped p-type silicon surface (resistivity <0.007 Ω cm) electron transfer between the silicon electrode and the ferrocene could proceed over several hundred redox cycles without any apparent oxidation of the underlying silicon^11^ and close to ideal surface electrochemistry.

**Fig. 1:**
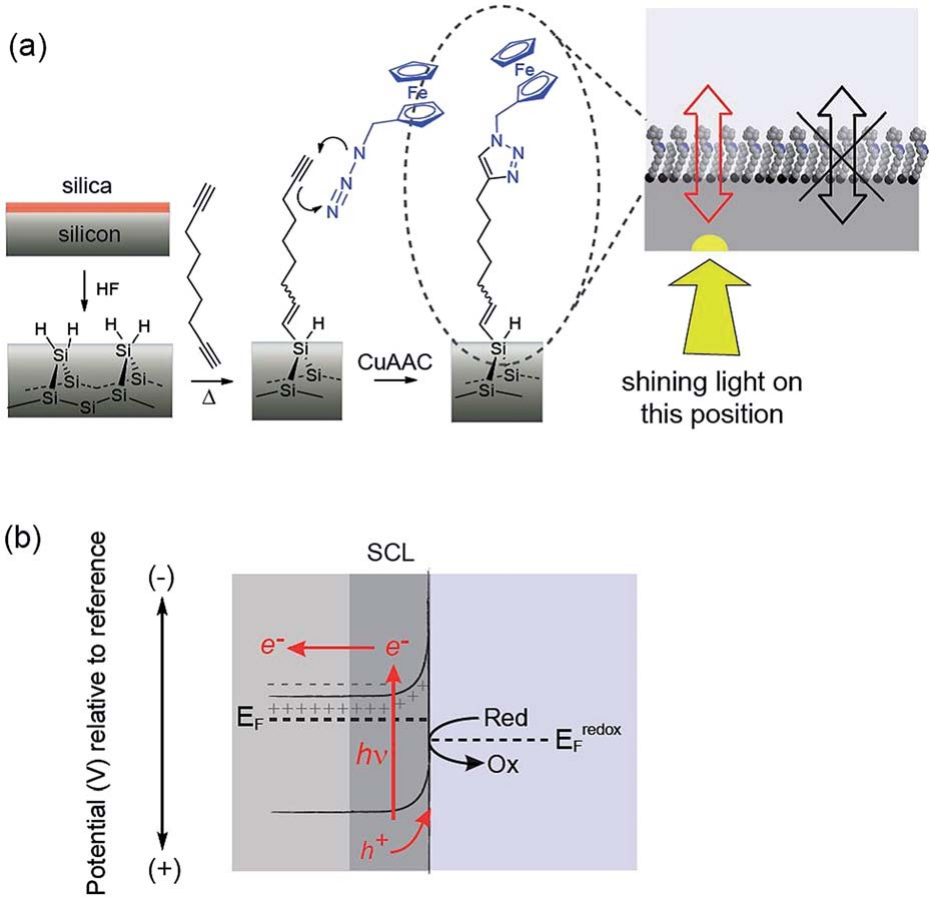
Schematics illustrating the concept of light activated electrochemistry and the general principle. (a) Shows the surface chemistry employed and a graphic suggesting electron transfer will only occur at a monolithic silicon surface where illuminated. (b) A diagram showing the working principles of how electrochemistry can be modulated on and off at a monolayer modified silicon surface using light. The semiconducting electrode is biased into depletion and the formation of space charge layer (SCL) impede electron transfer across the dark interface to insufficient charge carriers in this region. Illumination of the silicon results in an increase in charge carriers that allows appreciable electron transfer through the monolayer. The surface chemistry protects the non-oxide semiconductor from anodic degradation and allows electrochemistry with flowing currents to proceed only at the site of illumination.

It is combining silicon electrodes modified with this surface chemistry and light which is the key to the concept presented herein. By shining light on a completely flat unstructured silicon electrode, electrochemistry with flowing DC currents, *i.e.* faradaic electrochemistry, can be performed precisely at the site of illumination. In this way any location on an electrode surface can be interrogated electrochemically, with microscale resolution (*vide infra*), with only a single electrical connection at the periphery of the device. We call this new capability light activated electrochemistry. As depicted in Fig. 1, any part of a modified silicon surface can be locally activated to allow electrochemistry to be performed. Regions not illuminated remain electrochemically inactive. This greatly expands the scope of dynamic electrochemistry as (i) electrode arrays can be fabricated where each element on an array does not need a lead to make it independently addressable, and (ii) the position of each sensing/stimulating element is freed from geometric constraints. Furthermore, dynamic electrochemistry can be used to both read electrochemical information from a surface or to write electrochemically onto the surface.

The use of light to influence the electrochemistry of semiconductors is an established concept in photovoltaics and photocatalysis. For any semiconductor/electrolyte system, an applied potential exists at which the 10–1000 nm layer of the semiconductor adjacent to the electrolyte, is depleted of charge carriers.^13^ This region in contact with the electrolyte is known as the space-charge layer (SCL). When in depletion, the SCL is of high-resistance while the bulk of the doped semiconductor remains of lower resistance. For instance, n-type semiconductors are depleted at potentials positive of the device flat-band potential, *E*_FB_ (the applied potential at which there is no SCL). In the dark, these depleted electrodes have insufficient charge carriers available to transfer charge, and charge transfer is kinetically limited. This is the condition for the onset of electrochemistry at illuminated electrodes presented herein. In the dark no significant charge transfer occurs. When the depleted sample is irradiated with light of adequate energy however, a large number of charge carriers are generated. Electrons are excited to the conduction band with an equal number of holes in the valence band.^2^ That is, upon illumination for a depleted semiconductor, charge transfer is turned on. In the case of depleted n-type silicon electrodes modified with a ferrocene unit, electron transfer to the ferrocene can proceed only upon illumination because, only then, are their sufficient holes in the SCL of the electrode to accept electrons from the surface bound.

Herein the concept of spatially-resolved light activated electrochemistry on both n-type and p-type silicon is demonstrated by showing the localised electrodeposition of polypyrrole onto the electrode surface (writing) and by electrochemically detecting DNA hybridization (reading) from an array of DNA samples tethered on a macroscopic electrode.

## Experimental methods

### Chemicals

All chemicals, unless noted otherwise, were of analytical grade and used as received. Dichloromethane (DCM), light petroleum, ethanol, and ethyl acetate all were redistilled before use. Milli-Q water (∼18 MΩ cm) was used for preparing solutions, chemical reactions and surface cleaning procedures. Solvents used in the preparation and cleaning of silicon were hydrogen peroxide (30 wt% in water, semiconductor grade, Sigma-Aldrich), hydrofluoric acid (Riedel-de Haën, 48 wt% in water), sulphuric acid (semiconductor grade, Sigma). 1,8-Nonadiyne (98%, Sigma and 97%, Alfa-Aesar) was redistilled under reduced pressure from sodium borohydride (79 °C, 8–9 torr) and collected over activated molecular sieves (3 Å pore diameter, 10–20 mesh beads, dehydrated with indicator, Fluka) and stored under a high purity argon atmosphere (O_2_ < 5 ppb) prior to use. Hydroxymethylferrocene (97%, Alfa Aesar), and sodium azide (99.5%, Sigma-Aldrich) were used for the synthesis of azidomethylferrocene as described previously.^14^

Prime grade, single-side polished silicon wafers, 100-oriented (100 ± 0.5°), 500–550 μm thick were obtained from Siltronix Silicon Technologies (Archamps, France). n-Type (phosphorous), 8–12 Ω cm resistivity wafers were double-side polished and 175–225 μm thick. To further thin n-Si wafers to a thickness of 55 ± 6 μm (spatial resolution studies shown in Fig. 3) one side of the substrate was exposed to a 30% KOH aqueous solution at 40 °C for a 16 h period. The thickness of Si was measured for each sample by scanning electron microscopy imaging of the wafer cross-section.

### Assembly of monolayers of 1,8-nonadiyne on Si(100)

Si wafers were rinsed with DCM, dried under a stream of argon and immersed in hot (100 °C) piranha solution (1 vol 30% by mass aqueous hydrogen peroxide/3 vol sulphuric acid) for a 30 min period. Samples were then rinsed with copious amount of Milli-Q water before being transferred to an aqueous hydrofluoric acid solution (2.5%) for 90 s. The Si sample was then immediately transferred to degassed (through a minimum of five freeze–pump–thaw cycles) sample of 1,8-nonadiyne. The reaction mixture was kept under argon atmosphere and placed in an oil bath set at 165 °C for 3 h. After cooling down to room temperature, the acetylene-terminated Si sample was rinsed several times with DCM and rested in DCM at +4 °C under an argon blanket before being either analyzed or further reacted.

### ‘Click’ derivatization of acetylene-terminated Si(100) with organic azides

In a typical ‘click’ procedure for the preparation of ferrocene samples, to a reaction vial containing the alkyne-functionalized silicon surface, three components (i) azidomethylferrocene (8.67 mM, 2-propanol/water 1 : 1); (ii) copper(ii) sulphate pentahydrate (0.40 mM) and (iii) sodium ascorbate (20.19 mM) were added to a custom made reaction tube containing the alkyne-functionalized silicon surface. Reactions were carried out at a room temperature, in the dark and, excluding air from the reaction environment by applying a positive argon pressure and stopped after 45 min by removal of the modified sample from the reaction vessel. The prepared surface-bound [1,2,3]-triazoles were rinsed consecutively with copious amounts of water and ethanol, and then rested at room temperature for a 1 min period in a 0.5 M hydrochloric acid solution. Samples were then rinsed with copious amounts of water and ethanol before being either analyzed or further reacted. An analogous procedure was used for the ‘click’ of 2-(azidomethyl)anthracene-9,10-dione, in brief, to a reaction vial containing the alkyne surface were added (i) 2-(azidomethyl)anthracene-9,10-dione in degassed DMSO (7.59 mM in 10 mL DMSO); (ii) TMEDA (0.1 mM in 1.5 mL DMSO), and (iii) copper(i) bromide (0.04 mM). Air was excluded from the reaction tube and the reaction was continued in the dark at room temperature for 30 min and stopped by removing the silicon wafer from the reaction mixture. The anthraquinone bound surface was then rinsed thoroughly with redistilled ethanol and Milli-Q water. A final rinse with 0.5 M HCl was employed to remove any residual copper from the reaction, followed by rinsing in Milli-Q and ethanol.

Oligonucleotides-modified p-type Si electrodes for the electrochemical detection of DNA hybridization were prepared by dropping an alkyne-terminated surface onto 20 μL of a solution containing reagents for the click reaction of alkyne terminated single-strand DNA (ssDNA). This reaction mixture was made up of equal volumes of (i) 1,3-diazidopropane (800 μM in degassed DMSO/water/*t*-BuOH, 3 : 1 : 2 volume ratio), (ii) CuBr (490 μM) and TMEDA (1030 μM) in 3 : 1 DMSO/H_2_O, and (iii) 20 μM alkyne–DNA in 0.005 M phosphate buffer (K_2_HPO_4_/KH_2_PO_4_). The final concentration of alkyne DNA in reaction medium was 6.67 μM. The reaction was carried out at room temperature in the dark, excluding oxygen from the reaction environment and stopped after 1 h rinsing the sample with ethanol, water and ethanol. To limit the non-specific adsorption of the target DNA strand ssDNA modified surfaces were incubated for ∼4 h in a solution containing 1% bovine serum albumin and 0.05% sodium dodecyl sulphate in 0.05 M phosphate buffer containing 0.13 M NaCl, pH 7.0. This was followed by rinsing the surfaces with 0.05 M phosphate buffer containing 0.13 M NaCl. To ensure of releasing the weakly bound bovine serum albumin, modified surfaces were incubated in 0.05 M phosphate buffer containing 0.13 M NaCl for 1.5 h. Hybridization of ssDNA modified surfaces with complementary, non-complementary or C–A mismatch DNA targets was carried out by incubating the modified surfaces in 30 μL of 20 μM target oligonucleotide solution in 0.05 M phosphate buffer containing 0.2 M NaCl, pH 7 for 2 h (as individual drops on discrete locations on the ssDNA modified sample).

Electrochemical measurements to detect hybridization were also performed in the presence of anthraquinone-2-sulfonic acid (AQMS) in the electrolyte. To do this, before hybridization, modified surfaces were incubated in a mixture containing 20 μM of target DNA and 25 μM of AQMS in 0.05 M phosphate buffer containing 0.2 M NaCl for 3 h in the electrochemical cell. This was then followed by rastering the light source across the DNA spots with the silicon surface biased at –550 mV *versus* Ag|AgCl| 3 M NaCl.

### Electrochemical and spectroscopic measurements

All electrochemical measurements were performed using a Teflon three electrode cell with the modified silicon as the working electrode, platinum mesh as the counter electrode, and Ag|AgCl| 3 M NaCl as the reference electrode. All potentials were indicated as *E*, and reported *versus* the reference electrode. Ohmic contact between the silicon substrate and a copper plate was achieved by rubbing a gallium indium eutectic onto the backside of the silicon electrodes. A gasket defined the geometric area of the working electrode to 24.62 mm^2^. Cyclic voltammetry and chronoamperometry were performed using a BAS 100B electrochemical analyzer (Bioanalytical Systems, Inc., W. Lafayette, IN) and impedance measurements were performed using a Solartron 1255B (Farnborough, UK) frequency response analyzer interfaced to a Solartron 1287 potentiostat/galvanostat module.

For experiments using scanning light sources a custom built set-up, as shown in Fig. S3 and S10 (ESI‡) was built. Lens cage systems, 16 mm and 30 mm were connected with each other to put a mirror, a convex lens and a collimator. Because of the optical path between sample and laser, a collimator for a beam diameter of 1.5 mm (240FC-B, Thorlabs) was used to minimize the divergence of the laser and the other end was connected with a single mode optical fibre which carried the laser from the diode. The collimated light traverses through a plano-convex lens (focal length of 50 mm, LA1213-A, Thorlabs) and then reflected by a mirror (protected silver) at 45° to the laser diode output beam to focus at the back of the Si and yield best possible resolution. A pigtailed laser diode (LM9LP, Thorlabs) was connected with a laser diode controller (ITC4001, Thorlabs) and a potentiostat was connected to the sample using crocodile clip. During the operation of the pigtailed laser diode, temperature control was used to stabilize the laser's power and wavelength, while also prolonging the life of the laser. The laser diode temperature was set to 25 °C. Two mechanical stepper motors (DC servo motor controller TDC001, Thorlabs) were used to drive single axis translation stages (25 mm, PT1/M-Z8, Thorlabs) controlling the *x*/*y* position of light pointer across the unstructured electrode surface (Si chip).

X-ray photoelectron spectroscopy measurements were performed using an ESCALAB 220iXL spectrometer with a monochromatic Al Kα source (1486.6 eV). The pressure of the operating chamber was below 10^–9^ mbar and spectra were recorded in normal emission. The spot size was ∼1 mm^2^. The incidence angle was set to 58° to the analyzer lens. The resolution of the spectrometer is *ca.* 0.6 eV as measured from the Ag 3d_5/2_ signal (full width half maximum, FWHM) with a 20 eV pass energy.^11^ Survey scans were carried out over 1300–0 eV range with a 1.0 eV step size, a 100 ms dwell time and an analyzer pass energy of 100 eV. High-resolution scans were run with 0.1 eV step size, dwell time of 100 ms and the analyzer pass energy set to 20 eV. The background of the spectra was subtracted using the Shirley routine. The spectra were fitted with a convolution of Lorentzian and Gaussian profiles. The energies are termed as binding energies in eV and referenced to the C 1s signal (corrected to 285.0 eV).

## Results

There are several key steps to realizing the concept of light activated electrochemistry, and enabling dynamic electrochemistry to be performed anywhere on a monolithic electrode surface, at multiple locations in sequence. These are first being able to modify the silicon surface so it can sustain faradaic electrochemistry without being oxidized in the process, second to show under what conditions light can be used to switch electrochemistry on and off, third to show it can be performed anywhere on an electrode surface and finally show that it can be used to generate a simple electrode array with only a single wire at its periphery. We will discuss each of these steps in turn.

**Fig. 2:**
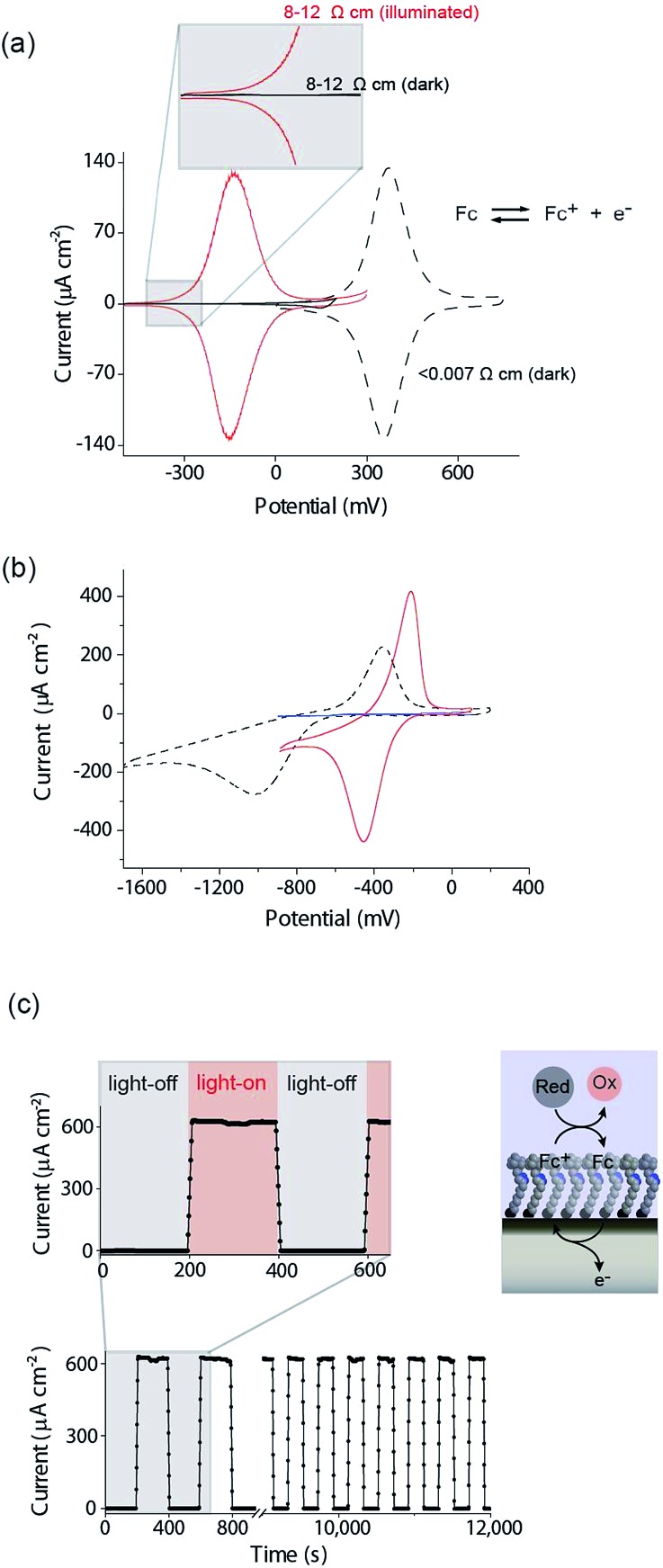
(a) A cyclic voltammogram (CV) (dash black line) in aqueous 1.0 M HClO_4_ at highly doped (<0.007 Ω cm, dopant concentration, *N*_A_, >8 × 10^18^ cm^–3^) p-Si electrodes that behave electrochemically similar to metals. A CV of a poorly doped n-Si electrode (8–12 Ω cm, *N*_D_, ∼4.5 × 10^14^ cm^–3^) in the dark (solid black line) and light (solid red line) at 642 nm, ∼300 mW cm^–2^. (b) CVs (dash black line) at highly doped p-Si electrodes (<0.003 Ω cm, dopant concentration, *N*_A_ ∼ 1 × 10^19^ cm^–3^) and poorly doped p-Si electrode (substrate: 1–10 Ω cm, *N*_A_, ∼8 × 10^14^ cm^–3^; illumination: 642 nm, ∼300 mW cm^–2^) to a surface bound anthraquinone at pH 10.1 in the dark (solid blue line) and light (solid red line). (c) Amperometric trace demonstrating the switching on and off of heterogeneous catalysis promoted by surface-bound ferrocene from an n-Si electrode to an aqueous ferrocyanide solution. The n-Si electrode is posed at +0.2 V *versus* Ag|AgCl| 3 M NaCl and 1 mM ferrocyanide [Fe(CN)_6_]^4–^ in solution recycles the ferricinium back to ferrocene with modulation of the illumination (0.1 M KNO_3_ at pH 7; substrate: 8–12 Ω cm; illumination: 527 nm, ∼1.77 mW cm^–2^, modulation is 200 s ON and 200 s OFF).

The modification of Si(100) surfaces with 1,8-nonadiyne and the subsequent click reaction with azidomethylferrocene have been described in detail previously.^11,15^ XPS characterisation of the modified silicon surfaces are shown in the ESI (Fig. S1 and S2‡). The important diagnostic feature for the quality of the surface modification is the Si 2p narrow scans where the absence of any peak between 101 and 104 eV after the formation of the monolayer from 1,8-nonadiyne and after the copper(i)-catalyzed azide–alkyne cycloaddition reaction. This is because a peak at 101–104 eV is indicative of silicon oxide and the absence of any peaks means there is no appreciable oxide on the electrode surfaces. If such a condition is fulfilled then close to ideal ferrocene electrochemistry is observed, as shown in Fig. 2a for highly doped p-Si electrodes (<0.007 Ω cm, dopant concentration, *N*_A_, >8 × 10^18^ cm^–3^) in aqueous 1.0 M HClO_4_. The level and type of dopant in these electrodes are such that they behave electrochemically similar to metals. We have shown previously that these electrodes are resistant to the formation of any surface oxide over several hundred redox cycles.^11^

Equivalent results are obtained for redox reactions in the cathodic region for p-type Si(100) electrodes modified with anthraquinone derivatives (Fig. 2b, <0.003 Ω cm, dopant concentration, *N*_A_ ∼ 1.0 × 10^19^ cm^–3^). Taken together the results for these two monolayer systems show that well defined electrochemistry can be obtained for systems of formal potentials across a wide window.

We next turn attention to poorly doped modified silicon electrodes where the semiconducting behavior of the silicon becomes apparent. With poorly doped n-type silicon (dopant concentration, *N*_D_, ∼4.5 × 10^14^ cm^–3^, resistivity 8–12 Ω cm), in the dark, no faradaic electrochemistry to a surface bound redox species is observed as shown by the featureless CV in Fig. 2a. Under these conditions the n-type silicon is in depletion as its flat band potential, *E*_FB_, was measured to be –0.31 ± 0.06 V (Fig. S5 (ESI‡)). To allow electrochemistry to proceed at these surfaces, illumination from the back-side (the side not exposed to solution) generates charge carriers in the semi-conductor. These charge carriers diffuse into the SCL and allow electron transfer to the redox species to proceed (Fig. 2a) with well-defined ferrocene/ferricinium electrochemistry observed where the *E*_1/2_ is –140 mV *vs.* Ag|AgCl| 3 M NaCl, n-type silicon is used. Similar results can be achieved with p-type silicon but using an anthraquinone derivative as the redox active species with a redox potential cathodic of the *E*_FB_ as shown in Fig. 2b where the *E*_1/2_ of the anthraquinone is –370 mV *vs.* Ag|AgCl| 3 M NaCl at pH 10.1.

The n-type and p-type silicon electrodes being in depletion at the appropriate potentials of the redox species is confirmed using Mott–Schottky measurements of the electrode/electrolyte (Fig. S5 and S6 (ESI‡)). More visual evidence that the n-type silicon is in depletion comes from the negative shift in the *E*_1/2_ for the ferrocene redox couple at the illuminated semiconductor electrode compared with the highly doped silicon electrode in the dark. This contrathermodynamic potential shift was previously shown to occur with the silicon electrode in depletion.^16^

The ability of the light to switch the faradaic electrochemistry of the surface bound ferrocene on and off is shown by modulating the light source on and off (Fig. 2c). Note here the n-Si electrode is poised at +0.2 V *versus* Ag|AgCl| 3 M NaCl and the ferrocyanide in solution recycles the ferricinium back to ferrocene. Over 12 000 s of cycling, each surface ferrocene molecule is being turned over approximately 1.7 × 10^5^ times and yet there is no measurable loss of catalytic activity.

**Fig. 3:**
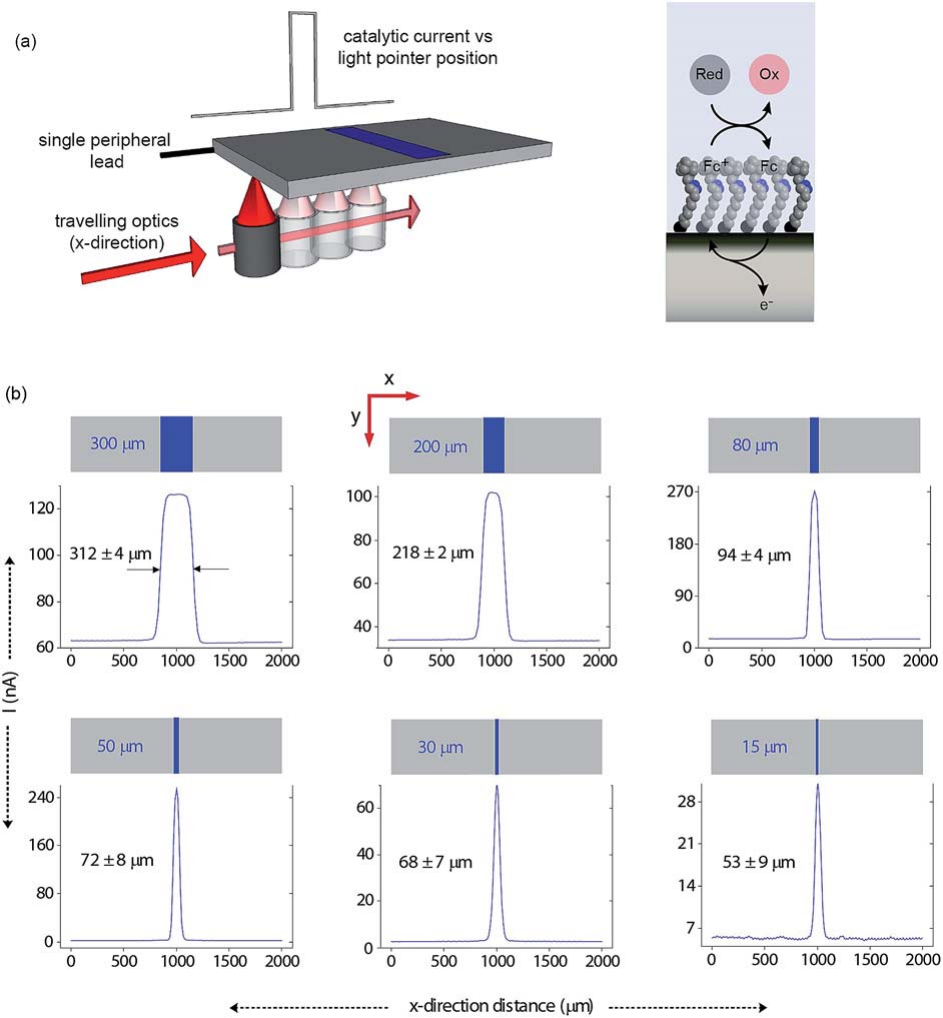
Direct measurements of the size of a 2D surface redox feature through a single peripheral connection. (a) A schematic of the experimental design where a travelling, collimated focused light beam (∼80 μm FWHM, *λ* = 642 nm, optical power is 0.1 mW, Fig. S4 in ESI‡) moves along the *x*-direction to illuminate the backside of a macroscopic n-type silicon electrode. On the upper side is exposed to 1 mM [Fe(CN)_6_]^4–^/100 mM KNO_3_ electrolyte solution and contains a patterned ferrocene feature (blue area) of known width. The faradaic current is recorded as a function of the light pointer position. (b) Current measurements for electroactive features on a monolithic electrode of 300 μm, 200 μm, 80 μm, 50 μm, 30 μm and 15 μm width. The minimal lateral spacing of adjacent features that can be resolved is close to the substrate thickness. Note that the adsorption depth for red radiation in silicon is <4 μm, and the substrate thickness is 55 ± 6 μm.

Having shown that light can activate the electrochemistry of surface bound species, in relation to using this phenomena to form electrode arrays, the next questions are can a faradaic process be spatially confined? To explore this issue we use classical microfabrication techniques to define covalent ferrocene feature of a precise geometry and size on a macroscopic nonadiyne-passivated n-Si electrodes (Fig. S8 (ESI‡)). This process results in silicon electrodes covalently decorated with ferrocene lines of known width, ranging from 300 μm down to 15 μm. The surface is then interrogated by poising the electrode at a potential where, under illumination, the electrocatalytic oxidation of [Fe(CN)_6_]^4–^ in solution, by surface tethered ferrocene units, occurs rapidly (turn-over number for the ferrocene/ferricinium couple is 26.5 s^–1^), see Fig. S7 (ESI‡). The focused light source (∼80 μm FWHM, 642 nm laser diode, Fig. S4 (ESI‡)) is rastered across the ferrocene line and the current recorded as a function of the travelling distance (Fig. 3a). As the light source moves across a ferrocene line, a rapid increase in current is observed. The width of the current trace (full width half maximum, *I*_FWHM_) tracks well the actual width of the ferrocene pattern (Fig. 3b). The *I*_FWHM_ was approximately 40 to 10 μm wider than the ferrocene features. The smallest features resolved for silicon of 55 ± 6 μm thick used herein was 53 ± 9 μm for the ferrocene line 15 μm wide. This broadening of the current peak is not unexpected as (i) photogenerated carriers can in fact diffuse over approximately the same distance laterally as they do in a direction normal to the surface^17^ (Fig. S9 (ESI‡) shows that with ∼200 μm thick silicon ∼300 μm spatial resolution is achieved), and (ii) light penetrating the wafer back side is isotropically scattered (adsorption depth for red radiation in silicon is <4 μm). Our experimental data shows that by addressing the device *via* backside illumination the achievable minimal lateral spacing between electrochemical features is close to the substrate thickness, an experimental limitation similar to the one encountered in LAPS.^18^ This comparison is important as with LAPS spatial resolutions as low as 15 μm can be achieved using topside illumination.^19^ Although achieving optimal spatial resolution is not the purpose of the current study, but rather to show that connecting electrodes with light can allow faradaic electrochemistry to be performed, the exploitation of the common photoactivation principle between light activated electrochemistry and LAPS suggests such similar spatial resolution can be achieved.

**Fig. 4:**
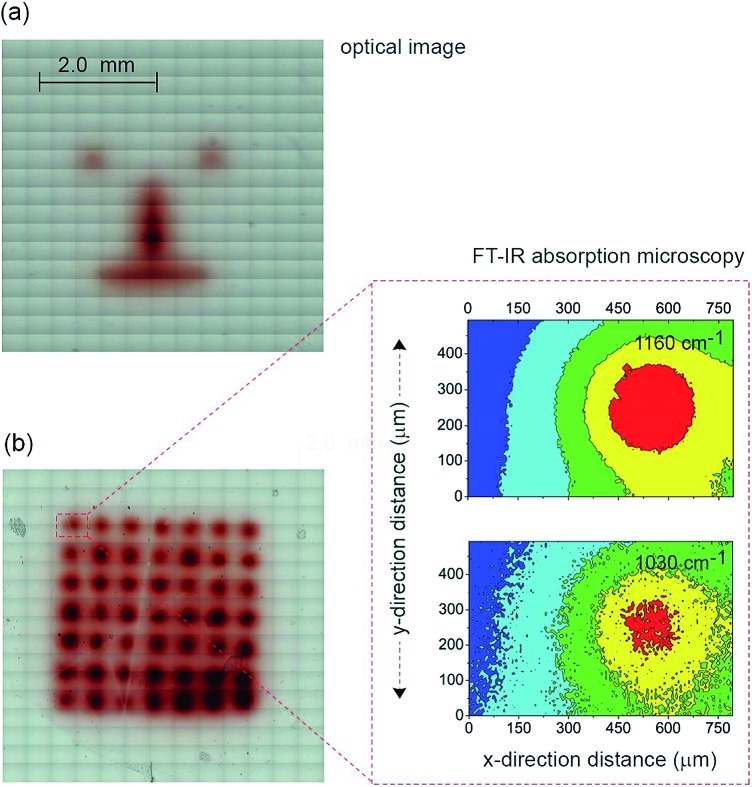
A demonstration of mask-free electrochemical writing: light-driven growth of conductive features on an insulating substrate. (a) Microscopy image of a “cartoon face”, achieved by light-assisted mask-free electrochemical growth of a conductive polypyrrole pattern on a n-type Si(100) electrode by scanning light at the back-side. (b) Optical microscopy and FT-IR absorption microscopy images of polypyrrole features electrodeposited at *x*- and *y*-distance intervals of 500 μm. Characteristics FT-IR bands confirm the chemistry and spatial arrangement of the polymer dots (1030 cm^–1^, C–H ring out-of-plane bending, C–C inter-ring out-of-plane bending, N–H ring out-of-plane bending; 1160 cm^–1^).

The next step in demonstrating the capability of light activated electrochemistry was to show the strategy allows electrochemistry to be performed anywhere on a monolithic surface. We demonstrate this by ‘writing’ conducting polymeric features at precise location on the surface of a passivated n-type silicon electrode. This was achieved using a 1,8-nonadiyne modified n-type silicon electrode (8–12 Ω cm resistivity, ∼200 μm thick, with *E*_FB_ of –0.34 ± 0.02 V) and electrodepositing polypyrrole from pyrrole monomers. Deposition of polypyrrole was achieved by poising the silicon electrode at +0.2 V (*versus* Ag|AgCl| 3 M NaCl) from a solution of pyrrole prepared at the concentration of 5.0 × 10^–1^ M in acetonitrile containing 1.0 × 10^–1^ M Bu_4_NClO_4_ under illumination (Fig. S11 (ESI‡)). The electrodeposition of the polymer was evident from the brownish-black film observable on the silicon electrode (Fig. 4a). In the absence of illumination no polypyrrole was deposited. Importantly, as the electrodeposition of the polymer in the n-type silicon electrodes occurs only when the electrode is illuminated, dots, lines and irregular shapes can be drawn (Fig. 4a). Confirmation of the deposition being polypyrrole comes from Fourier transform infrared microscopy images showing characteristics absorption bands at 780, 1030, and 1160 cm^–1^ (Fig. 4b).

**Fig. 5:**
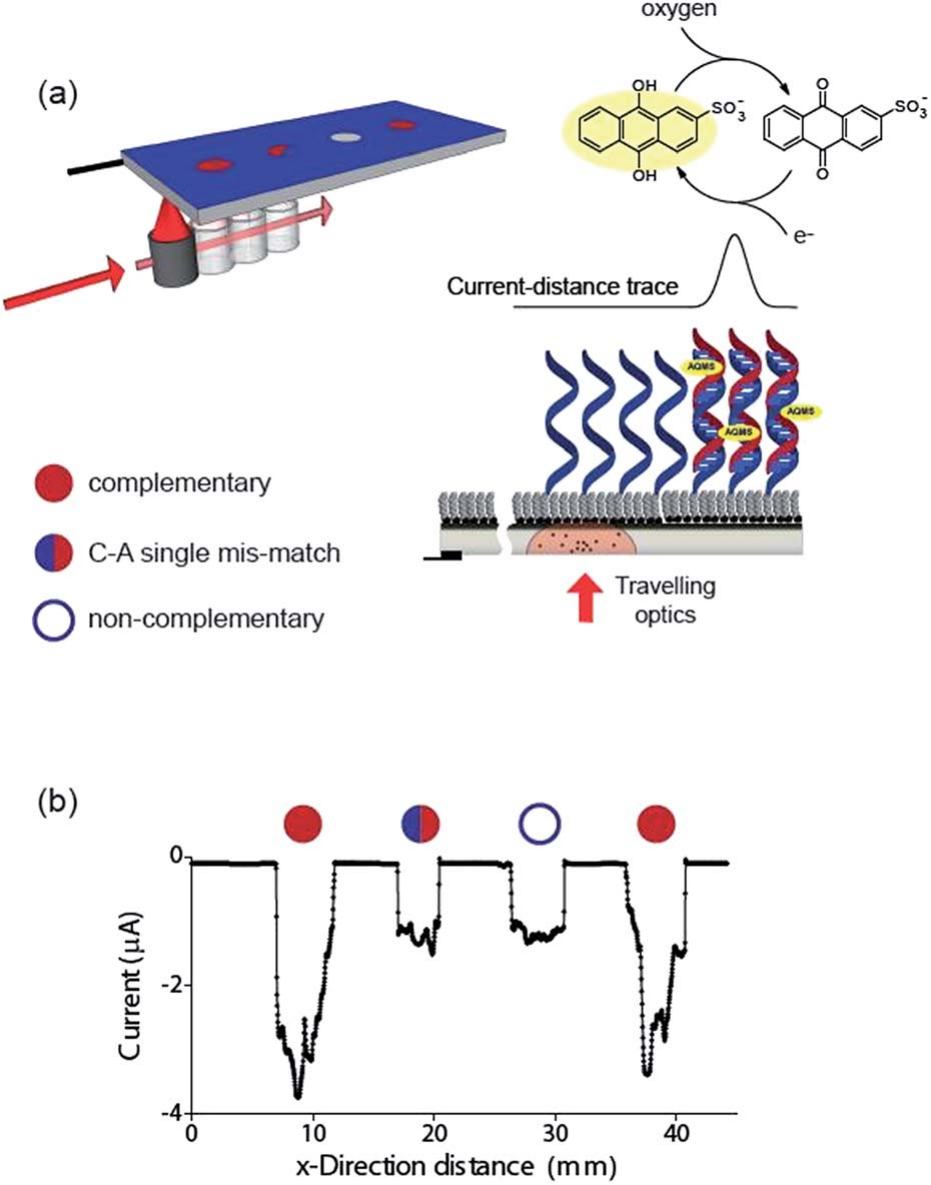
A demonstration that electrochemical information can be read from an array of DNA spots simply by scanning a light pointer across the surface. (a) Schematic of a chemically-modified p-type Si(100) photocathode device for spatially-resolved detection of DNA hybridization. Whether hybridization of target ssDNA in solution with surface-bound probe ssDNA occurs is determined by scanning the light source across the surface. If a duplex is formed, the redox species anthraquinone-2-sulfonic (AQMS) acid intercalates into the duplex and long range charge transfer can occur. The AQMS is recycled by dissolved oxygen in the buffer. (b) Electrochemical response of the modified silicon electrode poised at –0.55 V *versus* Ag|AgCl| 3 M NaCl as a function of the position of the light source across a four element array. At each position, a ssDNA probe sequence on the surface was exposed to target DNA that is complementary, possessing a single C–A base-pair mismatch, noncomplementary and complementary again to the surface probe. The solution contained 20 μM target DNA, 25 μM AQMS, 0.05 M phosphate buffer in 0.2 M NaCl.

Finally, the ability to use the light activated electrochemistry strategy to read electrochemical information from electrode arrays is demonstrated using electrochemical DNA arrays for detecting DNA hybridization. The detection of DNA hybridization was performed amperometrically using charge transfer through the DNA to an intercalated anthraquinone-2-sulfonic (AQMS) acid as depicted schematically in Fig. 5a. This is a strategy we have demonstrated previously that is capable of differentiating a complementary target strand of DNA from noncomplementary DNA in a very simple assay format on gold electrodes.^20,21^

It was first demonstrated that the long-range charge transfer strategy was possible on silicon electrodes. These experiments were performed on p-type Si(100) with (10–20 Ω cm) due to the reduction potential of AQMS occurring around –550 mV *versus* Ag|AgCl| 3 M NaCl. After modification with 1,8-nonadiyne, DNA with an alkyne linker at the 3′ end was coupled to the nonadiyne modified silicon using the ‘tandem click’ reaction strategy we reported previously.^22^ In this strategy 1,3-diazidopropane forms a link between the surface and the DNA (Scheme S3 (ESI‡)) *via* undergoing a CuAAC reaction with both the surface and the alkyne terminated DNA. Subsequently, the surface was incubated in bovine serum albumin, to reduce non-specific adsorption of target DNA,^23^ to give the final sensing surface.

To test that long range charge transfer through the DNA was occurring as was observed^20^ on gold electrodes, the sensing surface was exposed to target DNA in solution, incubated in 1 mM AQMS for 3 h before removing from the AQMS solution, and cyclic voltammetry being recorded (Fig. S13 (ESI‡)). In the absence of the light there is little visible faradaic electrochemistry (black line, Fig. S13 (ESI‡)) but upon illumination, for the complementary target sequence there is a strong reduction signal at –0.53 V (red line, Fig. S13 (ESI‡)) due to the reduction of the quinone moiety in AQMS. If only the single strand DNA modified surface is recorded in the presence of AQMS, the prominent faradaic peaks is not observed (blue line, Fig. S13 (ESI‡)).

Next we formed a simple array with a probe DNA attached at discrete locations on the electrode and each location exposed to a different target sequence: complementary, mismatched sequence and noncomplementary target DNA. The entire surface was poised at –0.55 V *versus* Ag|AgCl| 3 M NaCl and then the light source scanned across the different elements of the array in the presence of AQMS (25 μM) as depicted in the scheme in Fig. 5a. As the light scanned across a DNA spot there was an increase in the magnitude of the reduction current. Examples are shown in Fig. 5b for complementary, C–A mismatch and noncomplementary target DNA. As can be seen the reduction current was significantly greater for the complementary sequence compared with the single base-pair mismatch sequence and the noncomplementary sequence.

## Discussion and conclusion

In this paper we show that light can be used to form transient and spatially confined electrical connections to flat and unstructured electrodes. There are a number of conditions that must be fulfilled to achieve light activated electrochemistry. Firstly, the semiconducting electrode must operate in depletion. This means an n-type semiconductor for oxidation processes (Fig. 2a) or p-type conductors for reduction processes (Fig. 2b). In such cases there is no appreciable electron transfer across the dark semiconductor–monolayer–electrolyte interface. It is only upon illumination that the electrode is effectively connected at the site of illumination. The flow of DC currents across the active electrode elements can be of micrometer scale resolution (Fig. 3). Herein the spatial resolution was shown to be dependent on the thickness of the silicon wafer (Fig. S9 (ESI‡)) where the spatial resolution achievable when the device is illuminated from its backside is close to the substrate thickness. So, as shown in Fig. 3, for a 55 μm thick wafer, the experimental spatial resolution is 53 ± 9 μm.

It is prudent here to discuss the similarities between light activated electrochemistry and LAPS. Both methods employ semiconducting electrodes that are in depletion and rely on the generation of carriers upon exposure to light to allow the region illuminated to be addressed. Here the comparisons end. In LAPS, the electrode is isolated form the solution with an insulating layer and the interrogating light is modulated at high frequencies.^6–9^ In LAPS perturbations of the SCL by the ionic atmosphere in the solution account for changes in an AC photocurrent signal. No faradaic process or control of electrode kinetics can hence be achieved. In the approach discussed here, illumination of a region allows faradaic electrochemistry to occur in a specific region and allows detection of changes to the electrode kinetics (*e.g.* amperometric DNA hybridization sensing). This means dynamic electrochemistry can be performed anywhere on monolithic surface, with no requirement of light modulation and lock-in amplifiers. The light source can be continually scanned across a surface, as shown in both Fig. 3 and 5, and the change in current as a function of position can be recorded from a single wire connecting the electrode to an external circuit.

The ability to using light to localize flowing currents, with micron-scale resolution greatly expands the scope of dynamic electrochemistry as (i) an interface can be switched from effectively insulating to conductive by using a stimulus that is inherently clean and easy to deliver with high spatial and time control, (ii) the position of each sensing/stimulating element is freed from the constraints of a predetermined geometric arrangement of conductive pads, and (iii) electrode arrays can be fabricated with high density, as each element on an array does not need a lead to make it independently addressable. The first two of these advantages is illustrated in the paper by showing that materials can be electrochemically written to the surface, *via* the electrodeposition of polypyrrole. The full realization of the third feature is yet to be achieved, but the reading of electrochemical information from a four element DNA array is a first step in this direction. Whether DNA duplexes were formed at each spot was easily determined electrochemically by scanning the light source across the array, with a redox active intercalator in solution, such that an enhanced current was observed when the light scanned past a spot containing duplexes.
