# Supplementary material for: Connecting electrodes with light: one wire, many electrodes
Source: Chem Sci. 2015 Aug 28;6(12):6769–76. doi: 10.1039/c5sc03011k (PMC5508692; doi:10.1039/c5sc03011k)
Supplement: Supplementary file 1 [file SC-006-C5SC03011K-s001.pdf]

Electronic Supplementary Material (ESI) for Chemical Science.
This journal is © The Royal Society of Chemistry 2015

Supplementary Information for

Connecting Electrodes with Light: One Wire, Many Electrodes

Moinul H. Choudhury[1]†, Simone Ciampi[1]†, Ying Yang[1], Roya Tavallaie[1,2,3], Ying Zhu[1],

Leila Zarei[1], Vinicius R. Gonçales[1], J. Justin Gooding[1, 2, 3]

1. Synthetic Methods………… 02

1.1. Synthesis of 2-(azidomethyl)anthracene-9,10-dione………… 02

1.2. Synthesis of azidomethylferrocene………… 03

2. X-ray photoelectron spectroscopy (XPS)………… 04

2.1. Nonadiyne modified SAM on Si(100)………… 04

2.2. Ferrocene functionalized nonadiyne modified SAM on Si(100)………… 06

3. Laser set-up………… 09

4. Electrochemical measurements………… 10

4.1. Flat band potential measurements………… 10

4.2. Turn-over for the ferrocene/ferricenium couple………… 12

5. Patterning of the surface for spatial resolution measurements………… 14

5.1. Spatial resolution of redox features patterned on n-type Si(100) electrodes………… 15

6. Writing polypyrrole to a monolithic silicon surface………… 17

7. Reading electrochemical information of DNA arrays………… 19

8. References………… 25

1. Synthetic Methods

1.1. Synthesis of 2-(azidomethyl)anthracene-9,10-dione

The synthetic method was developed by Ciampi *et al* [1]. The synthetic process is shown in Scheme S1. *N*-Bromosuccinimide (NBS, 3.32 g, 18.7 mmol) and benzoyl peroxide (0.28 g, 1.2 mmol) were added to a stirring solution of 2-methylanthracene-9,10-dione (4.16 g, 18.7 mmol). The reflux was continued for ~ 21 h at room temperature with stirring under an argon atmosphere. After that, reaction mixture was cooled down in an ice-bath and filtered. The solid residue was washed with ice cold methanol (~ 100 mL). Filtration through celite and evaporation of the filtrate in vacuum produce off-white crude 2-(bromethyl)anthraquinone-9,10-dione, used for preparing 2-(azidomethyl)anthracene-9,10-dione without any further purification.

Sodium azide (4.5 g, 69.22 mmol) was added to 2-(bromethyl)anthraquinone-9,10-dione (500 mg, 1.66 mmol) in *N,N*-dimethylformamide (DMF, 20 mL) and Milli-Q H_2O with stirring at room temperature. The suspension was stirred under an argon atmosphere for ~ 20 h. The mixture was left in vacuum in a bath (temperature ~ 40 ºC, not exceed 60 ºC) to produce an off-white slurry. The slurry was then mixed with ethyl acetate (~ 50 mL). The suspension was filtrated and transferred to a separatory funnel, washed with Milli-Q H_2O (3 × 20 mL) and dried over Na_2SO_4 for ~ 3 h. Filtration and vacuum evaporation produced a pale yellow solid. Then column chromatography (toluene) produced desired off-white anthraquinone (390 mg, 78 %). ^{1}H (300 MHz, $CDCl_3$) δ: 8.31 (m, 4H), 7.81 (m, 3H), 4.59 (s, 2H); ^{13}C NMR (75.5 MHz, $CDCL_3$) δ:

182.83, 182.67, 142.19, 134.33, 134.25, 134.08, 133.95, 133.84, 133.47, 133.26, 133.18, 131.73, 128.06, 127.33, 127.31, 126.41, 54.08; IR (KBr, cm^{-1}): 2923, 2108, 1677, 1591, 1438, 1353, 1328, 1294, 1175.

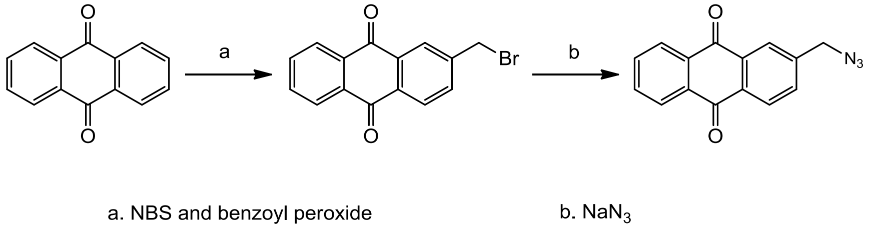

Scheme S1. Synthesis of 2-(azidomethyl)anthracene-9,10-dione.

1.2. Synthesis of azidomethylferrocene

Ciampi *et al*. reported the synthetic method to prepare azidomethylferrocene as shown in Scheme. S2 [2]. A solution of hydroxymethylferrocene (0.6 g, 2.78 mmol) and sodium azide (1.8 g, 27.69 mmol) in glacial acetic acid (~ 6 mL) was prepared. Then the solution was stirred at 50 ºC for 3 h under an argon atmosphere. The reaction mixture was diluted with 50 mL of DCM, and then organic phase was separated and washed with Milli-Q H_2O to remove the acid from the reaction mixture. The organic phase was washed with saturated $NaHCO_3$ (3 × 50 mL) and Milli-Q H_2O to remove the acid from the mixture completely, dried over Na_2SO_4, filtered and the filtrate dried *in vacuo* to yield an orange oil. The crude azide was purified by column chromatography (ethyl acetate : light petroleum = 1 : 1) to give orange solid (0.5 g, 83 %). ^{13}C NMR (75.5 MHz, $CDCL_3$)

δ: 77.46, 77.04, 76.61, 69.52, 50.99; IR (KBr, cm^{-1}): 3093, 2993, 2931, 2097, 2073, 1262, 1230.

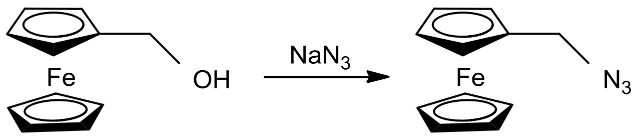

Scheme S2. Synthesis of azidomethylferrocene.

2. X-ray photoelectron spectroscopy (XPS)

2.1. Nonadiyne modified SAM on Si(100)

Surfaces of n-type Si(100) modified by thermally induced hydrosilylation reaction of 1,8-nonadiyne were characterised by XPS spectroscopy. The survey spectrums in Fig. S1a were carried out for 1350 to 0 eV. The survey scan indicates the presence of Si, C, and O, which correlates the presence of an organic monolayer on the silicon substrate [3]. Fig. S1b shows the narrow scan of the C 1s region, a broad signal with a mean binding energy of 285.0 eV. The dispersion value of the fitted function is 1.23 eV (FWHM). The large dispersion value is consistent with a peak resulting from carbon atoms bonded with carbon, hydrogen, and silicon bonded via either *sp*-, sp^2- or sp^3-hybridized. The peak at ~ 284 eV is due to the contribution of C-Si bonding. The narrow scan of Si 2p in Fig. S1c shows no significant oxide or suboxide silicon in the range of 102−104 eV. The absence

of any significant level of oxide indicates a well-defined monolayer which prevents the oxidation of the silicon substrate [4, 5]. The O 1s emission at ~ 532 eV might be due to the adsorbed oxygen.

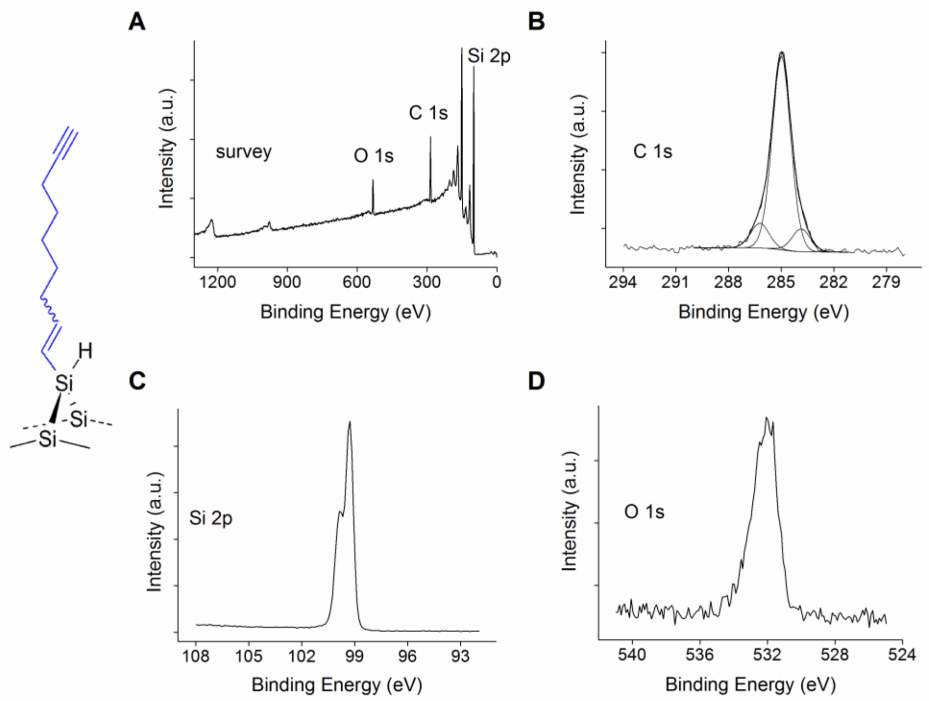

Fig. S1. XPS spectra of monolayers assembled from 1,8-nonadiyne on a hydrogen-terminated Si(100) sample. (**a**) Survey spectrum. (**b**) Narrow scan of the C 1s region. (**c**) High-resolution scan for the Si 2p region. Absent from the spectra is the signal associated with SiO_x species (102–104 eV). (**d**) Narrow scan of the O 1s region.

2.2. Ferrocene functionalized nonadiyne modified SAM on Si(100)

The successful formation of ferrocene-derivatized Si(100) with *in situ* generation of catalytically active Cu(I) species as evident from XPS spectra, is shown in Fig. S2. Fig. S2a shows nitrogen and iron-related emission peaks in the survey XPS spectra with Si, carbon, and oxygen signals. The detailed investigation of N 1s, Fe 2p and C 1s of the XP spectra in Fig. S2b-d indicates the successful triazole formation. From Fig. S2b, a broad signal for N 1s was observed at ~ 401 eV, that fits to two functions held at 1.4 eV apart (400.3 and 401.7 eV) with a 1.5:1 ratio of the integrated areas. The ratio and the signal peaks are correlated with peak positions reported by Ciampi *et al*. from the same group [5]. These two peaks at 400.3 and 401.7 eV may be related with the photoelectrons emitted from the nitrogen atoms of triazole moiety [3, 6]. The Fe 2p region in Fig. S2c showed the two major spin-orbit-split components Fe $2p_{3/2}$ and Fe $2p1_{/2}$ at 708.5 and 721.3 eV, respectively suggesting the presence of Fe(II) population with no evidence of Fe(III) species [7, 8]. The absence of Fe(III) species suggested that ferrocene was not oxidized to ferricenium under the reaction conditions [5]. Note that the absence of peaks at ~ 405 eV also indicates the presence of electron deficient nitrogen atoms in the azido moiety [5].

The narrow scan of C 1s in Fig. S2d shows the peak at 285 eV (FWHM of 1.37 eV) due to the aliphatic C-C and C atoms from the cyclopentadienyl rings of the ferrocene. The C 1s region was decomposed into two functions such as at 286.7 and 283.5 eV. The peak at 286.5 eV (FWHM of 1.44 eV) is related with the N bonded C (C–N) [3, 5]. The O 1s peak (Fig. S2e) at 532 eV might be due to the absorbed oxygen, also

observed for the nonadiyne modified Si [3]. The trace amount of Cu was also detected by the presence of Cu 2p peaks at 953.5 and 933.5 eV (20 eV apart from each other) as shown in Fig. S2f. Furthermore, the presence of F 1s at 686.3 eV is due to the etching by HF to remove the silicon oxide from Si (Fig. S2h). The narrow scan for Si 2p in Fig. S2g shows the absence of detectable emission related with silicon oxide species at (102-104) eV. The absence of peaks for silicon oxide indicates a high quality ferrocene-derivatized surface [3, 5, 9]. The conversion of nonadiyne modified Si to ferrocene-derivatized Si can be approximated by comparing the stoichiometry and % of the atomic composition from the XPS result. The coupling efficiency can be found from the ratio of the C 1s and N 1s regions and was found to be ~ 32 %, close to 40 % reported by the Ciampi *et al.* from the same group [5].

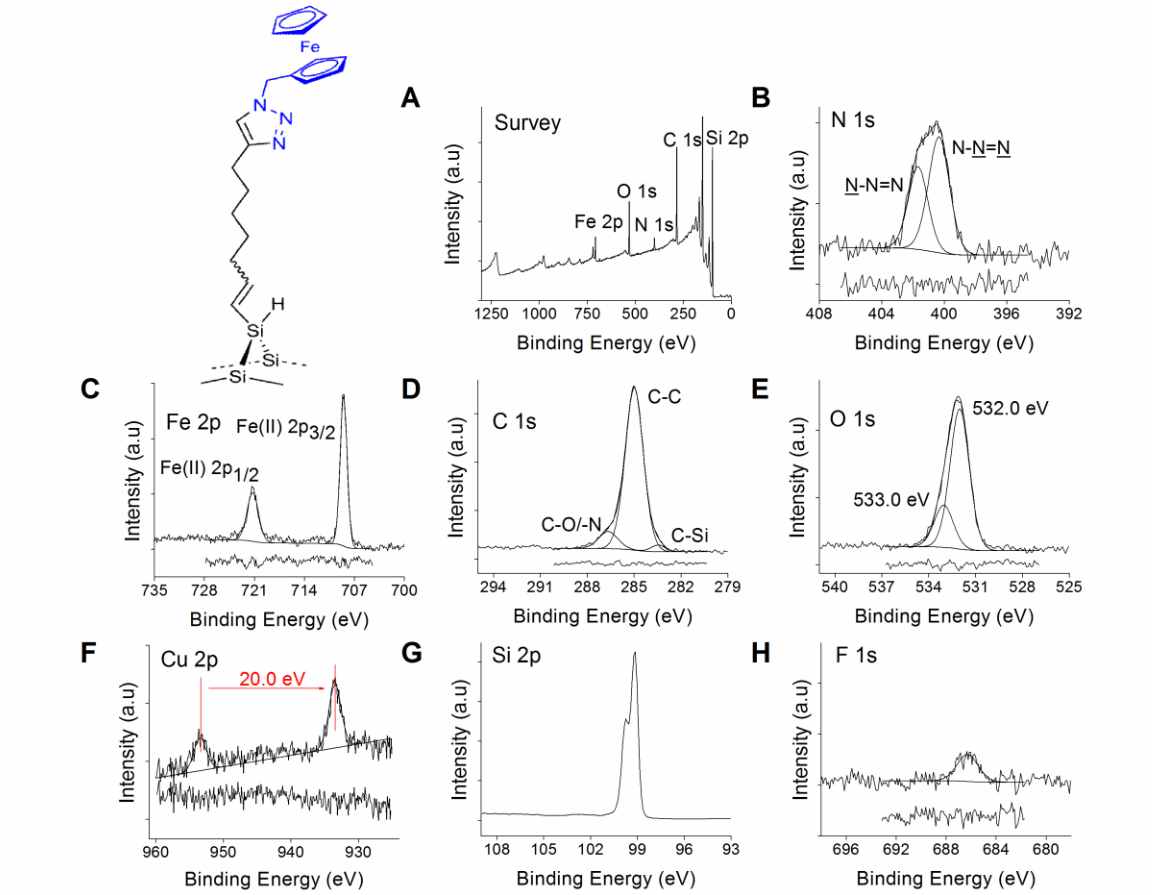

Fig. S2. XPS spectra of azidomethylferrocene-clicked nonadiyne modified Si(100) sample. (**a**) Survey spectrum. The narrow scan of the (**b**) N 1s region, (**c**) Fe 2p, (**d**) C 1s, (**e**) O 1s region, and (**f**) Cu 2p. (**g**) High-resolution scan for the Si 2p region. Absent from the spectra is the signal associated with SiO_x species (102–104 eV). (**h**) Narrow scan of the F 1s.

3. Laser set-up

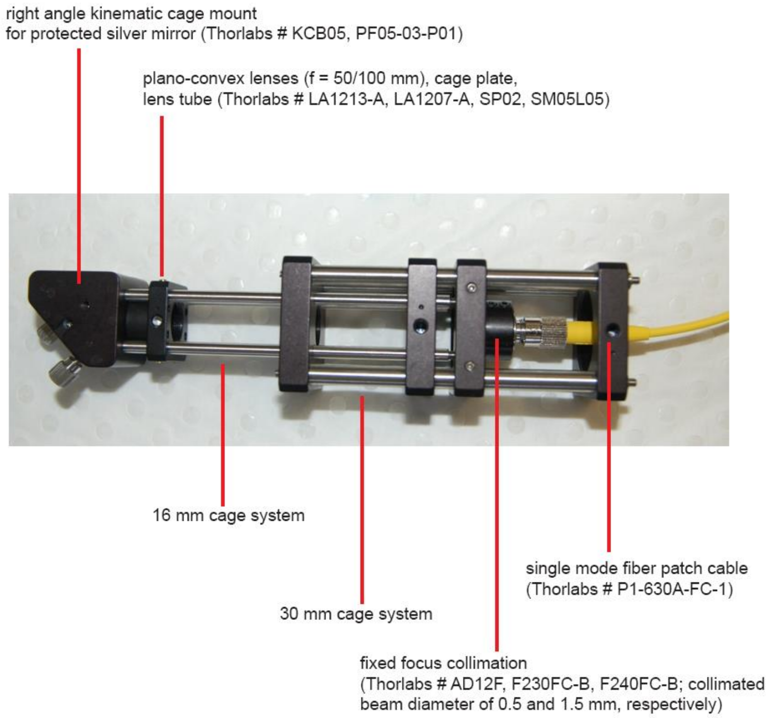

Fig. S3. Laser controller, laser diode, and the lens cage system which is placed underneath the sample to illuminate the Si chip from the bottom.

The laser beam profile (Fig. S4), defined as the FWHM was determined using a digital CCD camera (DCU224C, 1280 × 1024 Resolution, Color, USB 2.0, Thorlabs). For the experiments reported here the operating current was 40 mA which gave a FWHM of the laser beam on the backside of the silicon sample of 80 μm.

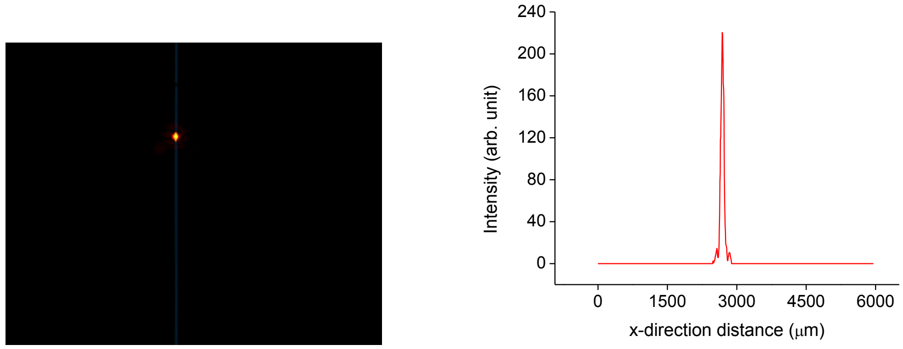

Fig. S4. Image of the continuous laser beam at a power of 0.1 mW and a wavelength of 642 nm. A collimator of 1.5 mm and a focal length of 100 mm were used to measure the size of the laser and the measured FWHM was 80 μm.

4. Electrochemical measurements

4.1. Flat band potential measurements

Impedance measurements were performed using a Solartron 1255B (Farnborough, UK) frequency response analyzer interfaced to a Solartron 1287 potentiostat/galvanostat module, and impedance data analyzed with ZView 3.1 and ZPlot software (Scribner Associates, Inc.). Flat band potential values were calculated from the extrapolation to $C-2 = 0$ of Mott–Schottky (M–S) plots [10, 11] ($C-2$ *vs*. E) with the measured capacitance C ($= 1/\omega[Y_T sin\theta]$) [12] calculated directly from the impedance magnitude Z_T ($Y_T = 1/Z_T$) and phase angle, θ, values.

The representative M−S plots for the nonadiyne modified Si are shown in Fig. S5a-c, as a function of frequency such as 10 kHz, 50 kHz and 100 kHz. A linear region was observed in the C^{-2}–E plot and the curve was fitted of this linear region. Fig. S5 shows the position of the E_{fb}, as determined by the intercept of the fitted line with the potential axis was − 0.31 ± 0.06 V for all frequencies in the presence of 0.1 M $NaClO_4$.

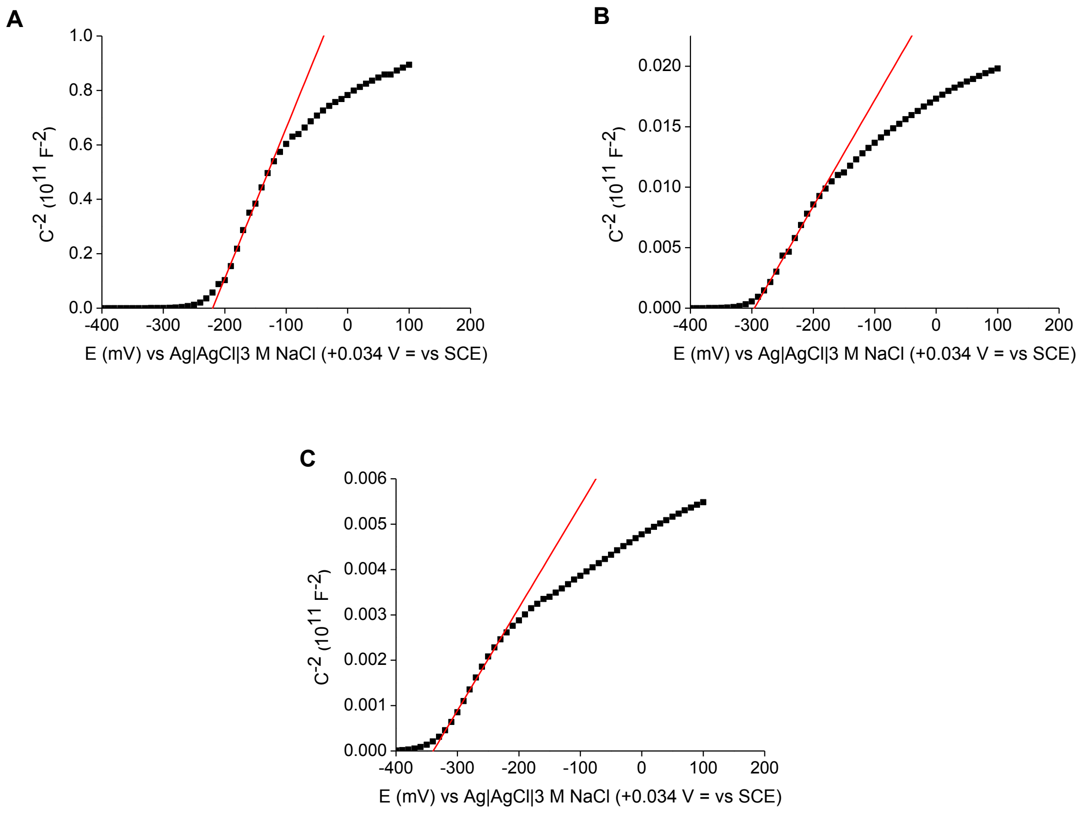

Fig. S5. M-S plot for monolayers of 1,8-nonadiyne on poorly-doped n-type Si(100) substrate (8-12 Ωcm) in the presence of 0.1 M $NaClO_4$. The solid red line (−) was plotted according to the M-S model. The frequency of the ac perturbations were (**a**) 10 kHz, (**b**) 50 kHz, and (**c**) 100 kHz. The impedance measurements were performed in the dark.

The M−S plots for the nonadiyne modified p-type Si(100) is shown in Fig. S6 at a frequency of 10 kHz. The flat band potential, E_{FB}, of the p-type Si is ~ − 0.482 V.

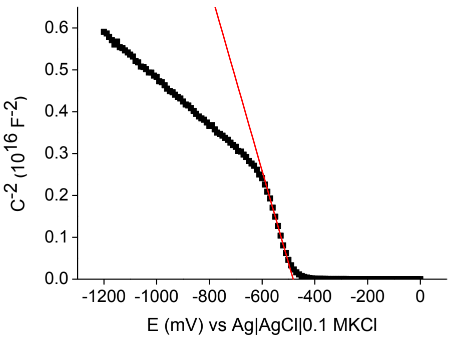

Fig. S6. M-S plot for monolayers of 1,8-nonadiyne on poorly-doped p-type Si(100) substrate (1-10 Ω cm) in the presence of buffer solution at pH 10. The solid red line (▬) was plotted according to the M-S model. The frequency of the ac perturbations were 10 kHz. The impedance measurements were performed in the dark.

4.2. Turn-over for the ferrocene/ferricenium couple

When the Si is biased at a potential of $E > E_{FB}$, under illumination, the photoanodic current is observed as ~ 600.0 μA due to the surface tethered ferrocene mediated charge transfer with $[Fe(CN)_6]^{4-}$, on the other hand, in the dark, the current is ~ 0.0 μA, which indicates that Si is in depletion. The rapid change in current under

illumination indicates the steady-state is reached within ~ (10-15) s. By knowing the surface confined ferrocene as shown in Fig. S7a and by integrating the amperometric trace as shown in Fig. S7b, the turn-over for the ferrocene/ferricenium is calculated.

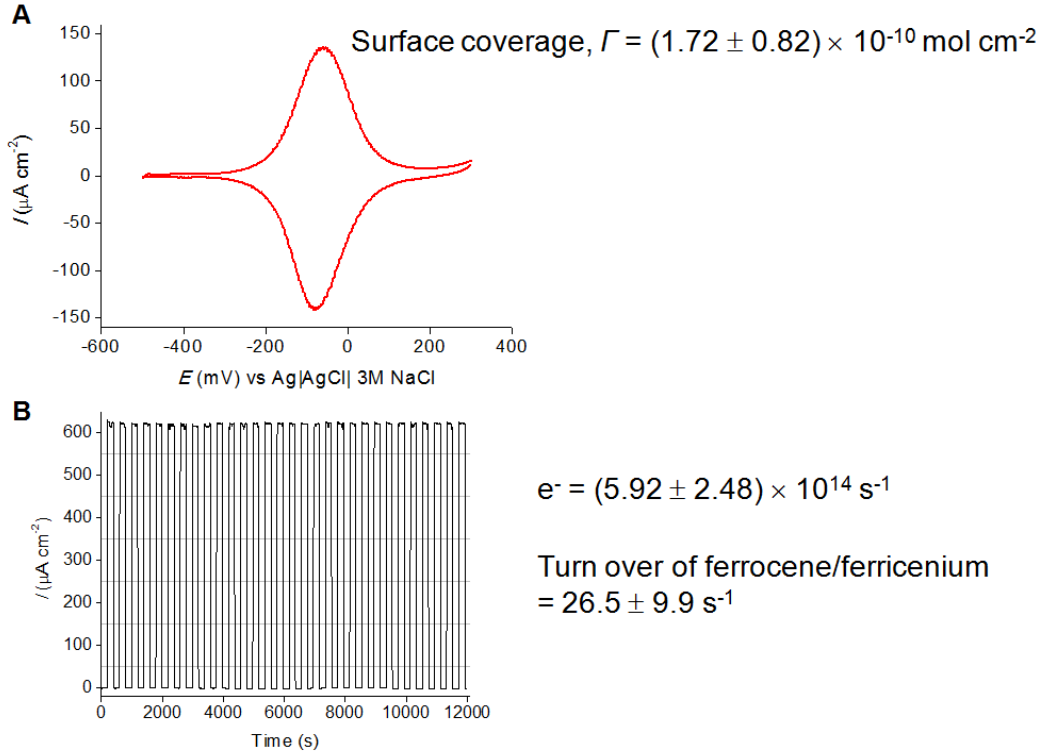

Fig. S7. (**a**) Surface coverage is calculated from the cyclic voltammogram (CV) under illumination. (**b**) Amperometric trace as a function of time demonstrating the surface tethered ferrocene-mediated charge transfer from an illuminated n-Si electrode to an aqueous solution of $Fe(CN)_6^{4-}$ (0.1 M KNO_3 buffered at pH 7; substrate: 8–12 Ω cm; illumination: 527 nm, ~1.77 mW cm^{-2}). From the surface coverage and integration of the I-t trace, the turn-over number for the ferrocene/ferricenium couple is 26.5 s^{-1}. There is a ~ 5 % decreases in current after 6000 s under light.

5. Patterning of the surface for spatial resolution measurements

Fig. S8 outlines how specific patterns for subsequent click reaction to allow the spatial resolution to be determined. The nonadiyne modified Si was spin-coated with SU-8 2007 photoresist (Microchem) at 3,000 rpm for 30 s. The sample was then baked on a hot plate at 65 °C for 2 min followed by 3 min at 95 °C. To form patterns the SU-8 photoresist was exposed to UV light for 22 s under a photomask with the desired pattern (Quintel Q6000 Mask Aligner) followed by repeating the baking process. The sample was then developed by immersing the wafer in SU-8 developer (Microchem) for 2 min, rinsed in 100% isopropyl alcohol and dried under nitrogen flow. The SU-8 photoresist in regions not exposed to UV light was then removed, resulting in the formation photoresist patterns. Subsequently azidomethylferrocene was 'clicked' onto the exposed regions to determine the spatial resolution.

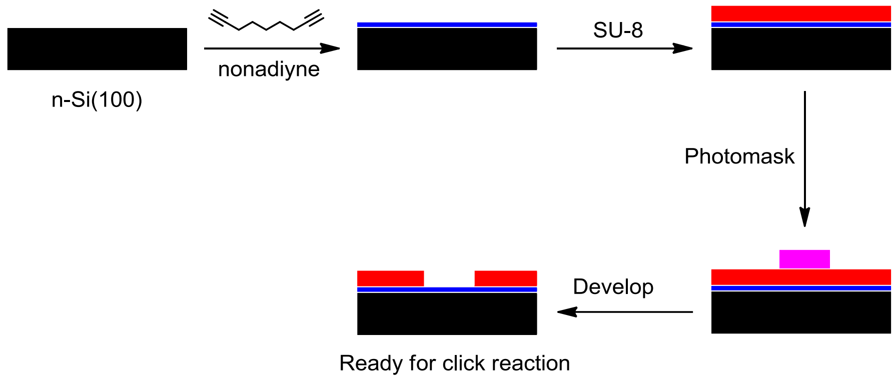

Fig. S8. Patterning of surfaces with photoresist for click reaction.

5.1. Spatial resolution of redox features patterned on n-type Si(100) electrodes

The electrochemical read-outs of a redox feature of known width (Fig. S9). The spatial information expressed as the FWHM of each current peak (current/distance trace) depend on the thickness of the silicon wafer (backside illumination). For a 200 μm thick wafer the readout of features sizes were found to be 349 ± 24 (A), 276 ± 26 (B), 296 ± 18 (C), and 297 ± 29 μm (D) for the actual feature of 300, 200, 80 and 50 μm, respectively.

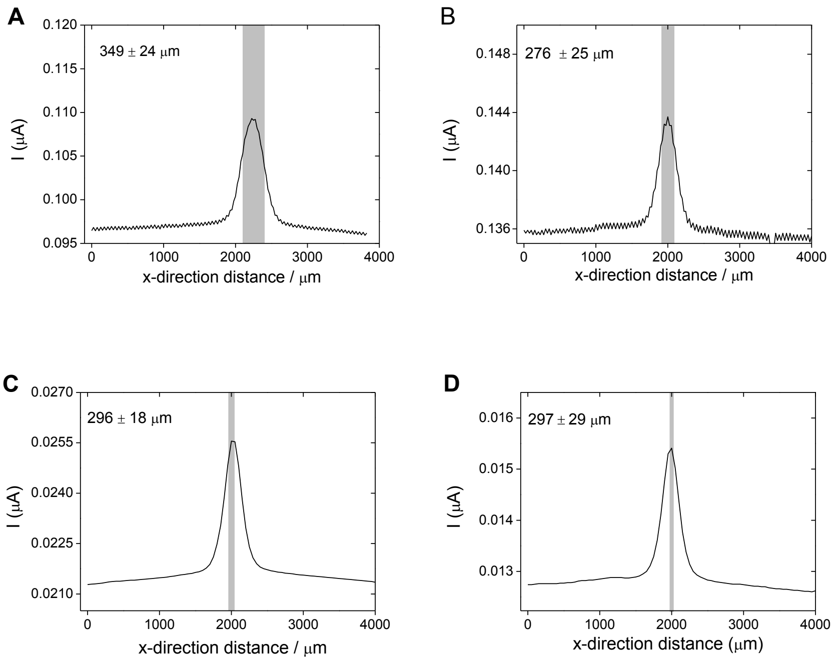

Fig. S9. Light-addressable read-outs of the feature size ranging from 300 (a) to 50 μm (d) with a Si thickness of ~ 200 μm. The spot size of light was 80 μm, operated at constant current of 0.1 mW and Si was biased at a voltage of 0.2 V Ag|AgCl|3M NaCl.

An improved resolution is shown in Fig. 3 of main text for silicon of 55 μm thickness compared with 200 μm thick silicon.

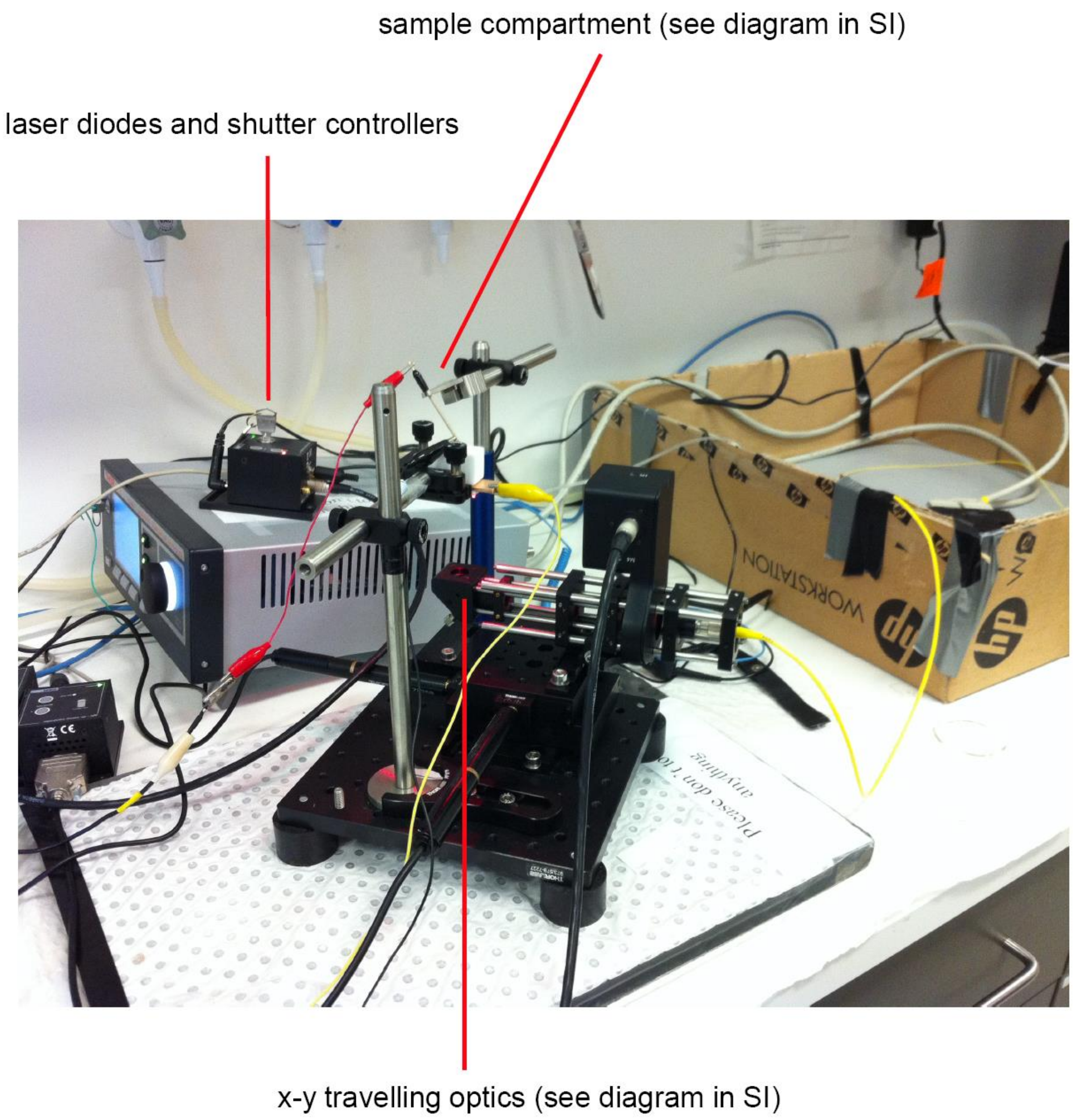

Fig. S10. Experimental set-up for the travelling light-pointer used the experiments of Fig. S9 and Fig. 3 of main text.

6. Writing polypyrrole to a monolithic silicon surface

The black line in the Fig. S11b is for the CV of the nonadiyne modified Si after the attempt to deposit polypyrrole in the dark for 300 s. No faradic polypyrrole electrochemistry is observed. In contrast, the CV (red line) after n-Si was poised at +0.2 V (*versus* Ag|AgCl|3M NaCl) and exposed to pyrrole for 300 s under illumination. The CV shows prominent oxidation and reduction of polypyrrole indicating under illumination polypyrrole was deposited on the nonadiyne modified silicon electrode. Reduction and oxidation peaks were observed at approximately – 0.31 and – 0.034 V vs. Ag|AgCl|3M NaCl, respectively and stable to cycling between – 0.7 and 0.3V in CH_3CN. Bard and co-workers reported broad reduction and oxidation peaks for polypyrrole on tantalum (Ta) at – 0.35 and – 0.0 V vs. SCE [13] and Yeu et al. mentioned cathodic and anodic peaks of polypyrrole films at – 0.45 and – 0.04 V, respectively [14] which is correlated with our observation.

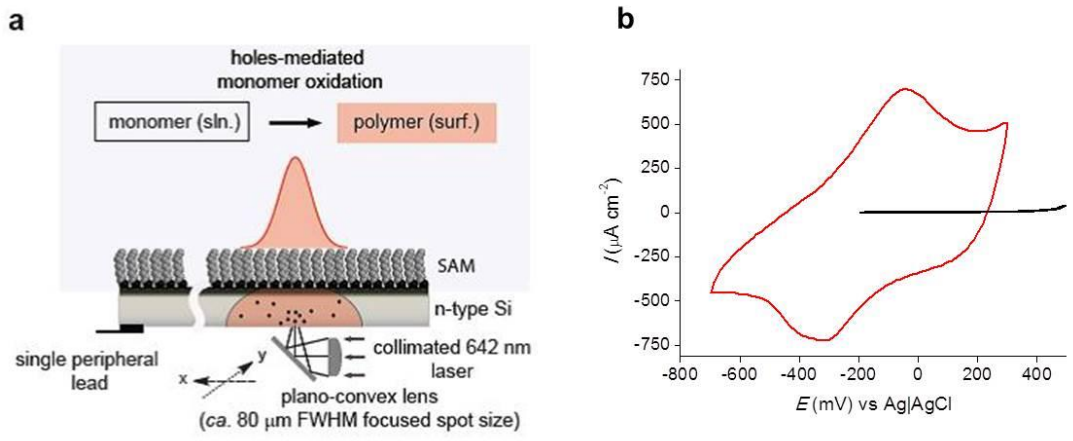

Fig. S11. A demonstration of mask-free electrochemical writing: light-driven growth of conductive features on an insulating substrate. (**a**) Experimental set-up for the light-assisted mask-free electrochemical growth of a conductive polypyrrole pattern on a n-type Si(100) electrode by scanning light at the back-side. (b) CV of the polypyrrole, deposited interface on 1,8-nonadiyne modified Si(100). The experiments were performed in the dark, (black line) represents the CV of non-structured nonadiyne modified Si before polymer deposition, (red line) represents the CV of polypyrrole deposited Si. The whole unstructured, nonadiyne modified Si(100) electrode was illuminated from the top with a white light to deposit polypyrrole from 0.5 M pyrrole in CH_3CN with the presence of 0.1 M Bu_4NClO_4. The light used for depositing polypyrrole on the modified Si was a halogen lamp (EJA halogen lamp, 21 V, 120W, Osram, Australia). During the polypyrrole deposition under illumination, the Si was biased at 0.2 V vs. Ag|AgCl|3M NaCl.

7. Reading electrochemical information of DNA arrays

The DNA modified interfaces were prepared as shown in Scheme S3.

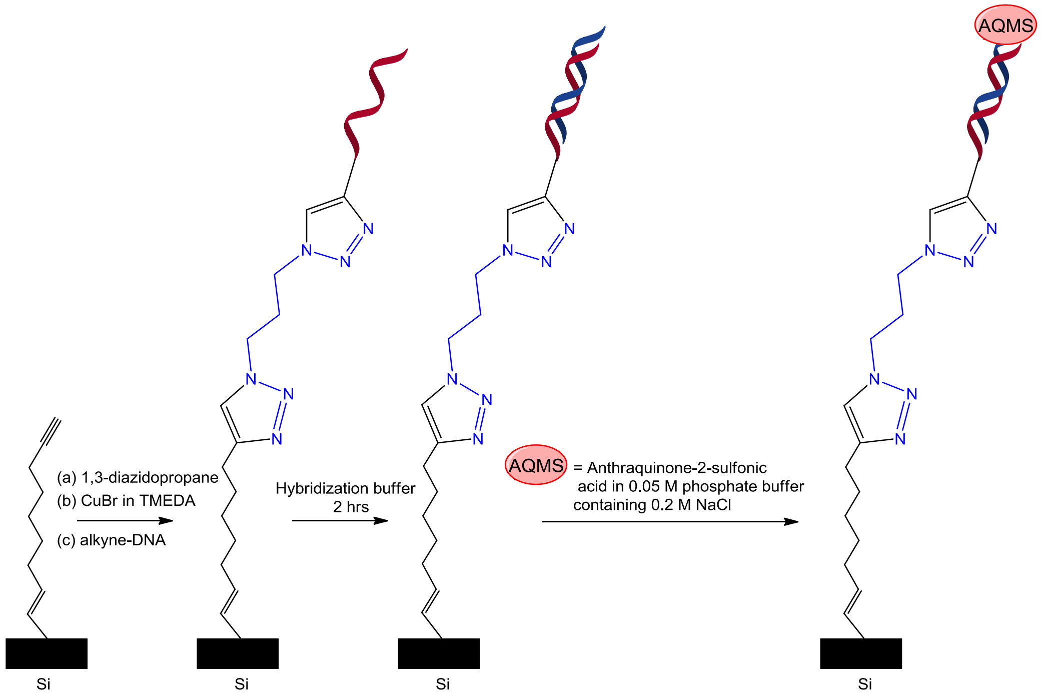

Scheme S3. The fabrication of the DNA selective interface where probe DNA is attached to the silicon surface *via* the 'tandem click' [15] reaction. Thereafter target DNA is hybridization onto the surface in a solution that contains hybridization buffer, followed by incubation in AQMS for 3 h.

The immobilisation buffer for clicking alkyne-DNA on nonadiyne modified Si surfaces consists of 0.05 M K_2HPO_4/KH_2PO_4, 0.13 M NaCl (pH 7.0). Furthermore, the

hybridization buffer contains 0.05 M K_2HPO_4/KH_2PO_4 in 0.2 M NaCl (pH 7.0). The DNA sequences used in this study are presented in Table S1.

Table S1. DNA sequences used in this study. Note there is one probe sequence and different target sequences. Hence in array experiments different targets are exposed to the surface at different locations.

Name	Sequence
Alkyne-DNA (probe-DNA)	5'- GGG GCA GTG CCT CAC AAC CT - 3'-alkyne
Complementary DNA target to probe-DNA (DNA 2)	5' - AGG TTG TGA GGC ACT GCC CC - 3'
Noncomplementary DNA (DNA 3)	5' - TCC AAC ACT CCG TGA CGG GG - 3'
C-A mismatch DNA target (DNA 4) to probe-DNA	5' - AGG TTG TGA GGA ACT GCC CC - 3'

Electrochemical measurements were performed using a custom designed electrochemical cell that had 6 openings at the base PTFE plate thorough which light can illuminate the Si from the bottom (Fig. S12). In addition, the PTFE upper part of the electrochemical cell has also 6 openings through which Si can be in contact with

electrolyte. The Si chip on a copper plate is placed in between the base plate and upper part of the cell.

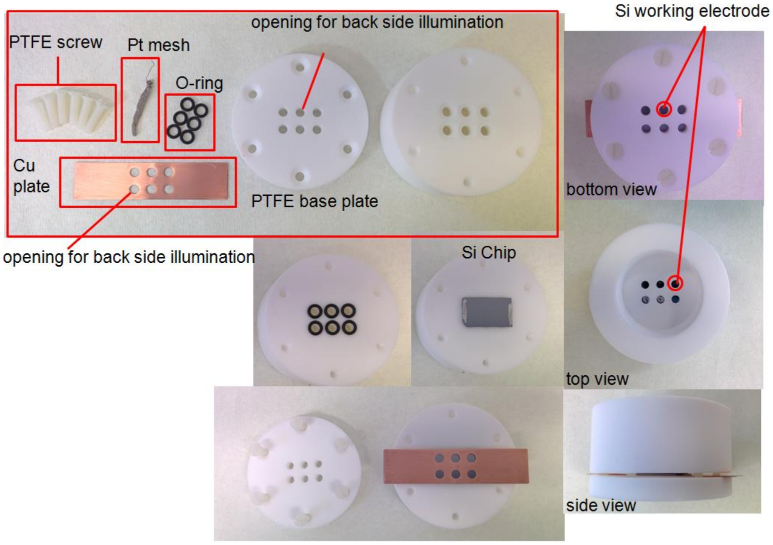

Fig. S12. The electrochemical cell used for electrochemical study, clicking DNA on the nonadiyne modified surface and hybridization.

The DNA experiments were based on previous work by us where charge transfer *via* surface immobilised DNA duplexes to intercalated redox species was demonstrated [16-18] at gold electrodes. This was achieved using the redox species, AQMS which is electrostatically repelled from the electrode interface, being anionic, but can intercalate into the DNA duplexes. Upon intercalation, it was shown that pronounced anthraquinone electrochemistry could be observed which was sensitive to single base-pair mismatches [16, 17] or other disruptions to the base pair stacking [18].

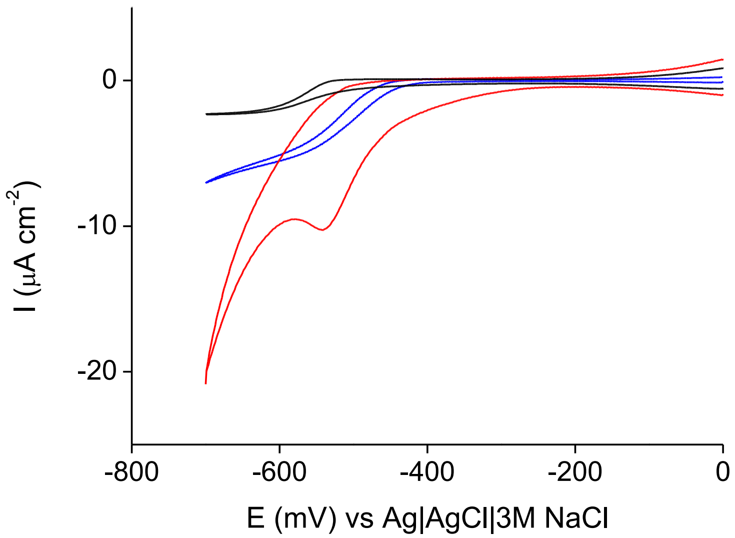

Fig. S13. CVs of the DNA modified p-type Si electrodes. (−) represents probe-DNA interface in the light and in the presence of AQMS in 0.05 M phosphate buffer containing 0.2 M NaCl. (−) represents probe-DNA interface after exposed to complementary target and AQMS in the Dark; and (−) the device exposed to a complementary DNA strand and AQMS in the light. The solution contained 0.05 M phosphate buffer in 0.2 M NaCl and scanned at a rate of 0.25 V s^{-1}. The target DNA analyte was at a 20 μM concentration in all cases.

Translating this type of DNA assay for light activated electrochemistry means the following criteria must be satisfied. Firstly, stable DNA interfaces must be formed, which we have recently demonstrated [14]. Secondly, a redox species must be surface bound to the monolayer such that there is appreciable electron transfer. Thirdly, the apparent formal potential of the redox species must be matched with silicon in depletion and finally there

must be a difference in electrochemistry between single strand probe DNA on the surface and DNA duplexes. As the redox potential of AQMS is around -550 mV *versus* Ag|AgCl|3M NaCl, p-type Si(100) can be used where it is hypothesized that intercalation of the AQMS into DNA duplexes will satisfy the criteria of a surface bound redox species with sufficient electronic coupling for charge transfer to proceed through the organic monolayer. Hence a difference in electrochemistry at surface DNA duplexes with intercalated AQMS is expected between the dark and light as shown in the CVs in Fig. S13. The fact that the AQMS does not intercalate if only single strand probe DNA is on the surface would therefore give a difference in Faradaic electrochemistry between ssDNA and dsDNA on the surface as observed (Fig. S14). These CVs demonstrate the charge transfer assay we developed previously can be combined with the light activated electrochemistry concept represented herein.

Next, arrays of spots of probe DNA were attached to the silicon surface and the laser light source scanned across the spots from the backside of the silicon. In the absence of AQMS there is almost no electrochemical signal (Fig. S14, −), while in the presence of AQMS a signal is observed in correspondence of DNA spot (−). As shown in Fig. 5b, a large electrochemical signal is observed in the present of DNA duplexes whereas the electrochemical signal for mismatch and noncomplementary spots were similar to the probe DNA spots.

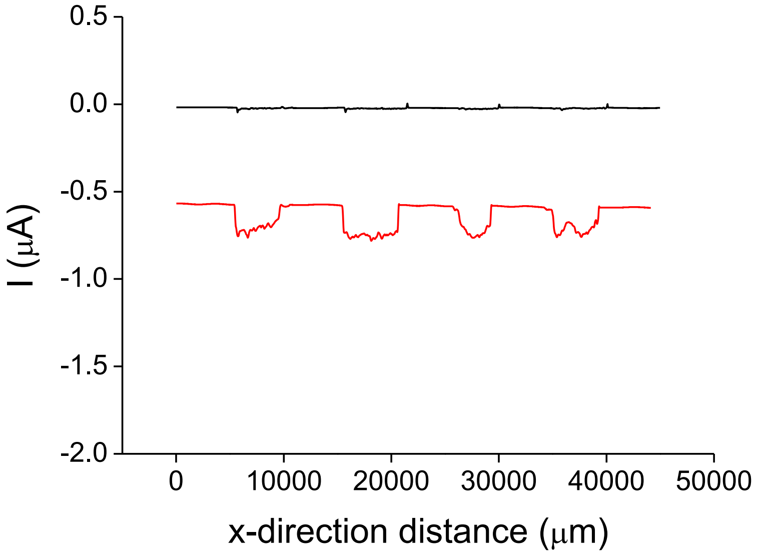

Fig. S14. Responses of the probe DNA arrays on the nonadiyne modified p-type Si electrode by scanning the laser light across the spots from the backside. After clicking probe DNA on the nonadiyne modified surface, the surface incubated in BSA solution for 4 h and then in 0.05 M Phosphate buffer in 0.13 M NaCl for 1.5 h. Subsequently, the interface (−) was scanned by laser light in 0.05 M phosphate buffer in 0.2 M NaCl. The red trace (−) represents the interface after exposed to 25 μM AQMS in 0.2 M NaCl and scanned in the same solution. It was found that the current in buffer increased when the surface exposed to AQMS.

8. References

1. S. Ciampi, *PhD Thesis*, 2009.
2. S. Ciampi, G. Le Saux, J. B. Harper and J. J. Gooding, *Electroanalysis*, 2008, **20**, 1513-1519.
3. S. Ciampi, T. Bocking, K. A. Kilian, M. James, J. B. Harper and J. J. Gooding, *Langmuir*, 2007, **23**, 9320-9329.
4. A. Salomon, T. Bocking, J. Gooding and D. Cahen, *Nano Letters*, 2006, **6**, 2873-2876.
5. S. Ciampi, P. K. Eggers, G. Le Saux, M. James, J. B. Harper and J. J. Gooding, *Langmuir*, 2009, **25**, 2530-2539.
6. R. D. Rohde, H. D. Agnew, W.-S. Yeo, R. C. Bailey and J. R. Heath, *J. Am. Chem. Soc.*, 2006, **128**, 9518-9525.
7. R. Zanoni, A. Aurora, F. Cattaruzza, C. Coluzza, E. A. Dalchiele, F. Decker, G. Di Santo, A. Flamini, L. Funari and A. G. Marrani, *Mater. Sci. Eng.*, 2006, **26**, 840-845.
8. N. Tajimi, H. Sano, K. Murase, K.-H. Lee and H. Sugimura, *Langmuir*, 2007, **23**, 3193-3198.
9. A. B. Sieval, R. Linke, H. Zuilhof and E. J. R. Sudhölter, *Adv. Mater.*, 2000, **12**, 1457-1460.
10. X. G. Zhang, *Electrochemistry of Silicon and its Oxide*, Kluwer/Plenum, New York, 2001.
11. P. Schmuki, H. Boehni and J. A. Bardwell, *J. Electrochem. Soc.* , 1995, **142**, 1705-1712.
12. H. G. L. Coster, T. C. Chilcott and A. C. F. Coster, *Bioelectrochem. Bioenerg.*, 1996, **40**, 79-98.
13. Z. Lin, Y. Takahashi, Y. Kitagawa, T. Umemura, H. Shiku and T. Matsue, *Anal. Chem.*, 2008, **80**, 6830-6833.
14. P. Michaels, M. T. Alam, S. Ciampi, W. Rouesnel, S. G. Parker, M. H. Choudhury and J. J. Gooding, *Chem. Comm.*, 2014, **50**, 7878-7880.
15. S. Ciampi, M. James, P. Michaels and J. J. Gooding, *Langmuir*, 2011, **27**, 6940-6949.
16. E. L. S. Wong and J. J. Gooding, *Anal. Chem.*, 2003, **75**, 3845-3852.
17. E. L. S. Wong and J. J. Gooding, *Anal. Chem.*, 2006, **78**, 2138-2144.
18. E. L. S. Wong and J. J. Gooding, *J. Am. Chem. Soc.*, 2007, **129**, 8950-+.
